# Regulating Glucose Metabolism Enzymes for Osteoporosis Therapy: Current and Future Approaches

**DOI:** 10.3390/ijms27104536

**Published:** 2026-05-18

**Authors:** Ziwen Zhang, Shuo Tian, Qian Li, Xiuwei Du, Linhui Wang, Na Li, Feng Zhao, Yanqiu Liu

**Affiliations:** 1 College of Traditional Chinese Medicine, Shandong University of Traditional Chinese Medicine, Jinan 250355, China; ziwenzhng@gmail.com (Z.Z.);; 2 Institute of Pharmacy (Institute of TCM Health Industrial Technology), Shandong University of Traditional Chinese Medicine, Jinan 250355, China; 3 Shandong Key Laboratory of Innovation and Application Research in Basic Theory of Traditional Chinese Medicine, Shandong University of Traditional Chinese Medicine, Jinan 250355, China; 4 Shandong Key Laboratory of Digital Traditional Chinese Medicine, Shandong University of Traditional Chinese Medicine, Jinan 250355, China

**Keywords:** osteoporosis, glucose metabolism, natural products, traditional Chinese medicine

## Abstract

Osteoporosis is a systemic skeletal disorder characterized by low bone mass, microarchitectural deterioration, and an increased risk of fracture. Its pathogenesis is closely associated with disturbances in energy metabolism, particularly glucose metabolic reprogramming in bone cells. Under osteoporotic conditions, the balance between osteoblasts and osteoclasts is disrupted, accompanied by impaired oxidative phosphorylation, dysregulated glycolysis, and reduced tricarboxylic acid cycle efficiency, ultimately leading to mitochondrial dysfunction. These metabolic alterations result in an insufficient energy supply and accelerate bone loss. Accordingly, the modulation of key enzymes involved in glucose metabolism has emerged as a promising therapeutic strategy. Strategies include the use of natural compounds, traditional Chinese medicine formulas, and specific inhibitors to modulate glucose metabolism processes and related pathways, thereby restoring cellular energy homeostasis and bone remodeling balance. This review summarizes pharmacological agents regulating glucose metabolism and proposes a hierarchical framework for therapeutic prioritization: first, inhibiting pathological glycolysis in osteoclasts (particularly via LDHA and PKM2). Second, restoring oxidative phosphorylation in osteoblasts (e.g., via COX I–V or ATP synthase). And third, employing multi-target traditional Chinese medicine formulas as complementary strategies. By establishing this cell-type-specific and pathway-specific hierarchy, the review aims to provide a theoretical basis for future research on metabolic interventions in bone diseases.

## 1. Introduction

Osteoporosis (OP) is a systemic skeletal disorder characterized by reduced bone mass, deterioration of bone microarchitecture, and an elevated risk of fragility fractures [1]. Epidemiological evidence indicates that OP affects approximately 200 million people worldwide and causes nearly 8.9 million fractures annually, equivalent to one fracture every three seconds among individuals older than 50 years. The prevalence is approximately 22.5% in women and 6.8% in men [2]. Consequently, OP has become a major public health concern that not only compromises quality of life but also imposes a substantial socioeconomic burden [3]. OP is generally classified as primary, secondary, or idiopathic; primary OP mainly includes postmenopausal and senile forms, whereas secondary OP may arise from metabolic disorders such as diabetes and hyperparathyroidism [4,5]. Despite their distinct etiologies, these subtypes share a common pathological feature, namely glucose metabolic reprogramming in bone cells, which disrupts osteoblast (OB)–osteoclast (OC) homeostasis and impairs bone remodeling [6,7,8,9].

As the principal effector cells of bone remodeling, OBs drive bone formation, whereas OCs mediate bone resorption. The balanced activities of these two cell types are essential for the maintenance of skeletal homeostasis. This process requires adenosine triphosphate (ATP) to support the synthesis of collagen and other matrix components [10]. Glucose serves as a critical source of carbon and energy [11]. Through glycolysis, the tricarboxylic acid (TCA) cycle, and oxidative phosphorylation (OXPHOS), glucose metabolism generates ATP to supply energy for OB differentiation and OC activation [12,13,14]. Complete oxidation of one molecule of glucose yields approximately 30–32 ATP molecules, thereby providing sufficient energy to sustain bone metabolic homeostasis [15].

Glucose metabolism in OP undergoes pathological reprogramming [16,17]. A hallmark of OBs in OP is excessive aerobic glycolysis, leading to lactate accumulation and local acidosis [18,19]. This pathological metabolic shift ultimately impairs cellular function and inhibits bone formation [20,21]. Upregulation of pyruvate dehydrogenase kinase (PDK) inhibits the pyruvate dehydrogenase complex (PDC), thereby limiting the conversion of pyruvate to acetyl-CoA [22,23]. This process often coincides with increased expression of glycolytic enzymes such as Hexokinase 2(HK) and lactate dehydrogenase A (LDHA), collectively shifting metabolism toward aerobic glycolysis [24]. As a result, the efficiency of the Tricarboxylic acid cycle (TCA cycle) is compromised, leading to reduced generation of reducing equivalents (NADH/FADH_2_), impaired OXPHOS, and diminished ATP generation. This is further associated with increased reactive oxygen species (ROS) production and mitochondrial damage [25,26]. These alterations reduce energy efficiency and promote OC differentiation and activation [27,28]. Collectively, metabolic reprogramming in both OBs and OCs disrupts bone remodeling balance and accelerates bone loss [29,30,31].

In traditional Chinese medicine (TCM), OP is primarily classified under the category of bone atrophy (wei). Its pathogenesis is attributed to aging and dysfunction of the spleen and kidneys, leading to impaired energy metabolism [32]. Kidney yang deficiency reduces warming and activation functions, whereas kidney yin deficiency diminishes nourishment to bone tissue, resulting in meridian obstruction and blood stasis [33]. Thus, OP is considered a condition of root deficiency with branch excess, fundamentally driven by energy metabolic disorders associated with visceral dysfunction. Accordingly, TCM interventions aim to regulate systemic energy metabolism and restore yin–yang balance, rather than merely supplementing calcium [34].

**Figure 1:**
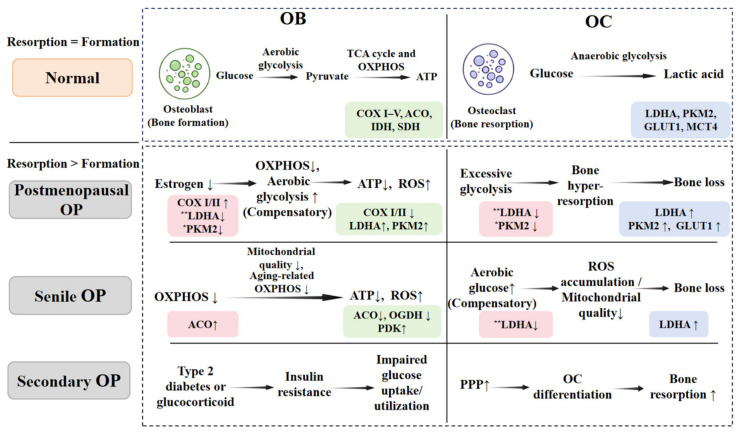
Glucose metabolic reprogramming characteristics of bone cells in different OP subtypes. Physiologically, OBs depend on OXPHOS and the TCA cycle for energy supply, while OCs rely on glycolysis. In senile, secondary, and postmenopausal OP, bone cells undergo distinct metabolic reprogramming featured by suppressed OXPHOS, compensatory elevated glycolysis, dysregulated key enzymes [e.g., LDHA, pyruvate kinase M2 (PKM2), glucose transporters 1/4 (GLUT 1/4), complexes (COX I–V), aconitase (ACO), isocitrate dehydrogenase (IDH), succinate dehydrogenase (SDH), PDK, α-ketoglutarate dehydrogenase (OGDH)], abnormal pentose phosphate pathway (PPP), reduced ATP, increased ROS, and mitochondrial dysfunction. These alterations disrupt bone remodeling balance, leading to excessive bone resorption and eventual bone loss. Red boxes indicate high-priority therapeutic targets [e.g., LDHA, PKM2, hypoxia-inducible factor 1-alpha (HIF-1α)], with the number of * corresponding to greater priority, whereas green and blue boxes represent osteoblastic OXPHOS markers and osteoclastic glycolytic markers, respectively.

Distinct metabolic alterations underlie different subtypes of OP [35]. In postmenopausal OP, estrogen deficiency induces mitochondrial dysfunction in OBs and enhances glycolytic reprogramming in OCs [36]. Senile OP is primarily associated with age-related mitochondrial decline, increased oxidative stress, and reduced metabolic flexibility [37,38]. In secondary OP, particularly that associated with diabetes, hyperglycemia impairs glucose uptake and utilization in bone cells, whereas recurrent hypoglycemia leads to insufficient energy supply [39,40]. Consequently, dysregulated glucose metabolism suppresses OB function while promoting OC activity, ultimately disrupting bone remodeling homeostasis [41] and exacerbating bone loss [42,43]. Therefore, strategies that enhance OXPHOS in OBs or suppress excessive glycolysis in OCs may represent effective therapeutic approaches (Figure 1).

Modulating key glucose metabolism enzymes represents a pleiotropic intervention that may reverse bone loss before structural deterioration occurs, positioning this approach as a promising therapeutic strategy for OP [44]. Natural compounds and TCM formulations have been extensively studied for their regulatory effects on cellular energy metabolism. These agents can modulate key enzymes and metabolic pathways, thereby restoring metabolic balance and providing a rationale for their therapeutic application. For example, metformin activates the AMP-activated protein kinase (AMPK) signaling pathway to regulate energy metabolism during bone remodeling [45]. Key enzymes such as LDHA and PDK directly influence OB differentiation and OC activation by regulating the balance between glycolysis and OXPHOS [46,47,48]. Several glycolysis-regulating inhibitors, including PKM2 inhibitor NCF and LDHA inhibitor FX11, are currently under preclinical investigation [49]. In addition, plant-derived compounds such as hydroxytyrosol, genistein, shikonin, and nuciferine (NCF), as well as TCM formulations, have shown potential to reverse metabolic reprogramming by modulating multiple metabolic enzymes, improving mitochondrial function, and regulating glycolytic flux [50].

Based on the distinct metabolic dependencies of OBs and OCs, we propose the following evidence-based therapeutic hierarchy for regulating glucose metabolism in OP: (1) Suppression of excessive OC glycolysis is the highest priority, as osteoclast-driven bone resorption is the dominant pathological driver across most OP subtypes. Within this tier, LDHA represents the most mechanistically central and actionable target, followed by PKM2. (2) Restoration of OB OXPHOS is a complementary priority, particularly via COX I–V or ATP synthase, to support bone formation. (3) Multi-component TCM formulas serve as holistic, multi-target strategies, especially when metabolic dysfunction involves both cell types or when subtype-specific mechanisms are unclear. This hierarchy is reflected in Figure 1 and the subsequent discussion, providing a consistent framework for evaluating current and future interventions.

Accordingly, this review systematically summarizes the roles of glucose metabolism-related enzymes in the pathogenesis of OP and highlights pharmacological strategies regulating these metabolic pathways, aiming to provide a theoretical basis for the development of novel metabolism-based anti-osteoporotic therapies.

## 2. Pharmacological Regulators of Glucose Metabolism Enzymes

### 2.1. OXPHOS

OXPHOS utilizes high-energy electrons from NADH and FADH_2_, transferred through the electron transport chain (ETC) COX I–IV, to establish a proton gradient that drives ATP synthesis via F_1_F_o_-ATP synthase [51,52,53,54,55]. The resulting proton motive force (PMF) is harnessed by F_1_F_o_-ATP synthase (also known as complex V), which couples the backflow of protons into the matrix with the phosphorylation of ADP to produce ATP, thereby achieving efficient conversion of proton potential energy into chemical energy [56,57]. Under osteoporotic conditions, OXPHOS is impaired. Cytochrome c, normally located in the intermembrane space, may leak into the cytoplasm through outer membrane pores or the mitochondrial permeability transition pore (mPTP) [58,59]. This leads to disruption of electron transfer between COX III and complex IV and reduces the efficiency of proton pumping. Concurrently, electrons are more prone to leak at sites such as COX I and III, where they can react with molecular oxygen to generate superoxide anions, resulting in excessive accumulation of ROS [60]. These alterations not only diminish the PMF and ATP synthesis but also contribute to the loss of mitochondrial membrane potential (MMP). Furthermore, they promote OB apoptosis and suppress bone formation [61,62].

#### 2.1.1. Drugs Regulating COX I-V

##### Hydroxytyrosol (HT)

HT, a natural polyphenolic compound with potent antioxidant properties, has been reported to exert protective effects against oxidative stress-induced OB dysfunction [63]. Mechanistically, HT improves OB viability and reduces apoptosis by attenuating ROS accumulation, restoring MMP, and enhancing ATP production. In addition, HT preserves mitochondrial respiratory chain function, including COX I–IV activity, and modulates Protein Kinase B (AKT)/Glycogen synthase kinase 3β (GSK3β) signaling pathways associated with mitochondrial integrity and OB survival [64].

However, several limitations should be considered. First, the current evidence is primarily derived from short-term in vitro studies, which do not adequately reflect the complex systemic and chronic nature of OP. Second, although the reported effective concentration (5 μM) falls within the upper range of human plasma levels (1–10 μM) [65,66], the pharmacokinetic stability and long-term exposure in vivo remain unclear. Third, the absence of in vivo validation and bone-specific functional endpoints, such as bone mineral density (BMD) or fracture risk, limits the translational relevance of these findings. Therefore, further studies using physiologically relevant models are required to establish its therapeutic potential.

##### Genistein

Genistein, a phytoestrogenic isoflavone, has been shown to enhance osteogenic activity and mitochondrial function in OBs [67]. Mechanistically, genistein promotes Estrogen receptor α (ERα) upregulation and mitochondrial translocation, increases COX I/II expression, and enhances ATP production, thereby supporting OXPHOS and cellular energy metabolism [68].

However, several critical limitations remain. First, the concentrations used in vitro (25–100 μM) substantially exceed clinically achievable plasma levels (1–5 μM) [69], raising concerns regarding translational feasibility and potential off-target effects. Second, although clinical trials have reported improvements in BMD at the lumbar spine and femoral neck, these studies are characterized by relatively small sample sizes and modest effect sizes, limiting the robustness of conclusions. Third, the absence of fracture outcome data, which represents the most clinically relevant endpoint in OP, further constrains the interpretation of therapeutic efficacy. Genistein is also widely used in clinical practice for the management of postmenopausal OP [70]. Additionally, increased gastrointestinal adverse events observed in clinical studies may affect tolerability [71,72]. Collectively, while genistein represents a promising metabolic regulator, its clinical utility requires confirmation in large-scale, well-designed trials incorporating fracture endpoints and long-term safety evaluation.

##### Estradiol

Estradiol plays a central role in bone metabolism through estrogen receptor-mediated mechanisms [73]. In OBs, estradiol induces ERα translocation to mitochondria, enhances COX I/II expression and activity, and increases ATP production, thereby promoting OXPHOS and osteogenic differentiation [74].

However, despite its well-established osteoprotective effects, several limitations restrict its clinical application. First, estradiol exerts systemic hormonal effects, including increased risks of breast cancer and thromboembolism, which limit its long-term therapeutic use. Second, the mechanistic evidence is largely derived from in vitro or preclinical studies, and the specific contribution of mitochondrial metabolic modulation to clinical outcomes remains insufficiently defined. Third, regulating upstream hormonal pathways may lack specificity, potentially affecting multiple tissues beyond the skeletal system. Therefore, future strategies should focus on selectively regulating downstream ERα-related metabolic pathways to improve safety and specificity.

##### α-Lipoic Acid (α-LA)

α-LA, a mitochondrial antioxidant, has been shown to improve OB viability, mineralization capacity, and mitochondrial function under conditions of oxidative stress. Mechanistically, α-LA enhances ATP production, reduces ROS accumulation, improves MMP, and activates phosphoinositide 3-kinase (PI3K)/AKT signaling, thereby contributing to mitochondrial protection and OB survival [75].

However, several important limitations should be noted. First, the experimental model relies on acute mitochondrial toxin-induced stress [e.g., antimycin A (AMA)], which does not adequately represent the chronic and multifactorial metabolic dysfunction observed in OP. Second, the findings are primarily based on in vitro studies, lacking in vivo validation and bone-specific outcome measures. Third, the relevance of these acute stress responses to long-term bone remodeling processes remains unclear. Consequently, the translational applicability of α-LA in OP therapy requires further validation in physiologically relevant models.

##### Er-Xian Decoction (EXD)

EXD is an empirical formula developed by Professor Zhang Bona at Shanghai University of TCM. The formulation consists of *Curculigo orchioides* (20 g), which warms and tonifies kidney yang, strengthens bones, and dispels dampness; *Morinda officinalis* (15 g), which similarly tonifies kidney yang and strengthens sinews and bones; *Anemarrhena asphodeloides* (10 g) and *Phellodendron chinense* (10 g), which nourish yin, drain fire, promote fluid production, and moisten dryness; *Angelica sinensis* (15 g), which warms and enriches the blood to tonify deficiency; and *Epimedium brevicornum* (20 g), which disperses and dispels dampness while warming the kidney and invigorating yang. When combined, these herbs achieve the effects of tonifying yang and nourishing yin. This formulation harmonizes the kidney’s qi, blood, yin, and yang, gradually restoring healthy qi, thereby alleviating symptoms such as cold pain in the lower back and weakness of the limbs [76]. It is characterized by a multi-component and multi-target mode of action that aims to restore systemic metabolic balance. In vitro studies demonstrate that EXD enhances OB proliferation, alkaline phosphatase (ALP) activity, and bone matrix mineralization, while increasing ATP synthase expression, suggesting improved OXPHOS. In OCs, EXD inhibits tartrate-resistant acid phosphatase (TRAP) activity and reduces bone resorption [77]. Although proteomic analyses suggest that EXD upregulates ATP synthase, the tested concentration range (50–200 μg/mL) does not establish a clear correlation with human plasma exposure, and the in vitro model fails to account for the metabolic transformation of herbal compounds by gut microbiota.

In a clinical study, 90 postmenopausal women (aged 50–75 years, and at least 1 year postmenopause) diagnosed with OP complicated with kidney yang deficiency syndrome were randomly assigned to two groups. The diagnostic criteria for OP were based on the Guidelines for the Diagnosis and Treatment of Primary Osteoporosis (2017) [78]. The diagnostic criteria for kidney yang deficiency syndrome were based on the Expert Consensus on the Prevention and Treatment of Primary Osteoporosis with Traditional Chinese Medicine (2020) [79]. However, several limitations constrain the interpretation of these findings. First, the in vitro concentration range (50–200 μg/mL) lacks clear correspondence to human plasma exposure, limiting dose translation. Second, in vitro models do not account for the complex metabolic transformation of herbal components by gut microbiota, which may substantially alter bioactivity. Third, clinical studies are predominantly open-label, with short treatment durations and absence of fracture endpoints, reducing the strength of evidence [76,80]. Additionally, the lack of standardized formulations and pharmacokinetic characterization further limits reproducibility and translational applicability. Therefore, while EXD demonstrates multi-target potential, rigorous standardization and high-quality clinical trials are required [81].

##### Zhuanggu Zhitong Formula (ZGZTF)

ZGZTF was originally documented in “*Ben Cao Jing Shu*” and evolved from the ancient formula Buguzhi Wan, which consists of *Psoraleae Fructus*, *Epimedii Folium*, *Lycii Fructus*, *Ligustri Lucidi Fructus*, *Drynariae Rhizoma*, *Cibotii Rhizoma*, *and Cyathulae Radix*. Based on the TCM theory of “tonifying the liver and kidney, strengthening tendons and bones”, ZGZTF is clinically used for primary OP, including postmenopausal and senile OP, to alleviate bone pain and restrict joint mobility [82]. It is commonly administered orally as a decoction or in capsule form for a treatment course of 3–6 months [83].

ZGZTF (13 weeks) increased femoral and lumbar BMD, restored bone mineral content (BMC), upregulated creatine kinase (CK) and ATP synthase expression, and modulated fructose bisphosphate aldolase and secretogranin II [84]. This study, however, is subject to certain limitations, notably the absence of detailed information in the source literature regarding the standard drug administration protocols. Furthermore, the lack of dose–response data and the use of non-standardized extracts hinder reproducibility.

ZGZTF achieved a total effective rate of 94.0%, improved low back pain and fatigue, and increased lumbar spine BMD (*p* < 0.05) compared to nilestriol. Mild gastrointestinal discomfort was reported [85]. However, the open-label design, short duration, and lack of fracture endpoints limit the strength of the conclusions.

ZGZTF combined with teriparatide [86], ibandronate [87], or raloxifene [88] further improved BMD, bone turnover markers, and quality of life compared to monotherapy, with faster symptom relief and fewer adverse events. Combination therapy also demonstrated faster symptom relief and a lower incidence of adverse events. Nevertheless, these studies are predominantly open-label and are not powered to assess fracture outcomes, limiting their clinical interpretability.

##### Qianggu Bao No. 1 (QGB-1)

QGB-1 is a clinically utilized TCM formula for the management of postmenopausal OP. It exhibits a multi-component, multi-target mode of action that regulates bone metabolic homeostasis [89]. The composition of QGB-1 was as follows: *Astragali Radix* (20 g), *Drynariae Rhizoma* (10 g), *Dipsaci Radix* (10 g), *Ligustri Lucidi Fructus* (10 g), *Rehmanniae Radix Praeparata* (15 g), *Cyathulae Radix* (9 g), *Salviae Miltiorrhizae Radix* (10 g), and *Atractylodis Macrocephalae Rhizoma* (10 g). In Ovariectomized (OVX) rats, QGB-1 (0.8 g/kg/d for 20 weeks) significantly increased femoral BMD and restored it to sham-operated levels, while modulating ATP synthase and PK isozyme expression [90].

Clinical studies further indicate that QGB-1, when combined with conventional anti-osteoporotic therapy, attenuates periprosthetic bone loss and improves BMD preservation without increasing the incidence of adverse events [91]. However, several limitations should be acknowledged. First, the clinical studies are characterized by small sample sizes and short follow-up durations, limiting generalizability. Second, the use of surrogate endpoints, such as periprosthetic BMD, does not fully capture clinically relevant outcomes such as fracture risk. Third, the lack of standardized formulations and pharmacokinetic data restricts reproducibility and mechanistic interpretation. Consequently, further large-scale, well-controlled studies are required (Table 1).

##### Sodium Butyrate (NaB)

NaB, a short-chain fatty acid, has been reported to exert beneficial effects on bone metabolism under metabolic stress conditions; however, these findings should be interpreted with caution due to model-specific and translational limitations. In a high-fat diet-induced model, NaB improves BMD and trabecular microarchitecture while contributing to the restoration of calcium metabolic balance. Mechanistically, NaB upregulates OXPHOS-related genes, including NAD4L and ATP synthase subunits, and activates nuclear factor erythroid 2-related factor 2 (Nrf2)/GSK-3β-mediated antioxidant signaling pathways. In vitro, NaB enhances OB viability, mineralization, MMP, and ATP production under oxidative stress conditions, accompanied by reduced ROS and malondialdehyde (MDA) levels and increased antioxidant enzyme activities such as superoxide dismutase (SOD) and catalase (CAT).

However, several important limitations should be considered. First, the high-fat diet model primarily reflects metabolic syndrome rather than canonical OP, which may confound the interpretation of bone-specific effects and limit disease relevance. Second, the mechanistic findings are largely correlative, and direct causal links between OXPHOS modulation and bone remodeling outcomes remain insufficiently established. Third, the in vitro experiments are based on acute oxidative stress conditions, which do not adequately recapitulate the chronic and multifactorial metabolic dysfunction characteristic of OP. Furthermore, the absence of standardized dosing and the use of percentage-based administration complicate dose translation to clinical settings. Additionally, the reduced efficacy observed at higher concentrations suggests a narrow therapeutic window, raising concerns regarding safety and clinical applicability. Collectively, these factors limit the translational potential of NaB and highlight the need for validation in physiologically relevant OP models with clinically meaningful endpoints [92].

##### Methyl-Piperidino-Pyrazole (MPP)

MPP is a highly selective ERα antagonist widely used in mechanistic studies of estrogen signaling. In OBs, MPP inhibits ERα mitochondrial translocation, leading to reduced COX I/II expression, decreased complex IV activity, and impaired ATP production, thereby suppressing OXPHOS. Functionally, MPP attenuates osteogenic differentiation, as reflected by decreased expression of bone morphogenetic protein 6 (BMP-6), type I collagen (COL1), and ALP, along with reduced mineralization capacity [74].

However, several limitations restrict its relevance in the context of OP therapy. First, MPP is primarily a pharmacological tool compound rather than a therapeutic agent, and its effects are mainly used to model estrogen deficiency rather than to provide direct therapeutic benefit. Second, the evidence is derived exclusively from in vitro systems, which lack the systemic hormonal regulation and complex bone microenvironment present in vivo, thereby limiting physiological relevance. Third, inhibition of ERα signaling may exert widespread effects across multiple tissues beyond bone, raising concerns regarding specificity and potential off-target effects. In addition, the mechanistic findings focus predominantly on mitochondrial endpoints without direct evaluation of bone-related functional outcomes, such as BMD or fracture risk. Therefore, MPP should be regarded primarily as an experimental probe for dissecting ERα-dependent metabolic pathways rather than a viable therapeutic candidate for OP.

### 2.2. Glycolysis

Glycolysis is a major pathway for cellular energy production and enables ATP generation even under hypoxic conditions [93,94]. HK catalyzes the phosphorylation of glucose to glucose-6-phosphate and serves as the first rate-limiting enzyme in glycolysis [95]. Phosphofructokinase-1 (PFK), the key rate-limiting enzyme in glycolysis, catalyzes the conversion of fructose-6-phosphate to fructose-1,6-bisphosphate [96]. Aldolase then cleaves fructose-1,6-bisphosphate into two three-carbon intermediates, glyceraldehyde-3-phosphate and dihydroxyacetone phosphate [97]. During the energy-yielding phase, glyceraldehyde-3-phosphate is oxidized to form 1,3-bisphosphoglycerate, which is subsequently converted by Phosphoglycerate kinase (PGK) to generate the first molecule of ATP through substrate-level phosphorylation [98]. Pyruvate kinase (PK), the third major rate-limiting enzyme, catalyzes the final substrate-level phosphorylation step, converting phosphoenolpyruvate to pyruvate and ATP [51,99]. Overall, glycolysis yields a net gain of 2 ATP molecules and NADH per molecule of glucose [100,101]. Under anaerobic conditions, LDHA reduces pyruvate to lactate, thereby regenerating NAD^+^ and sustaining glycolytic flux [102,103].

In OP, mitochondrial dysfunction and oxidative stress in OBs directly disrupt glycolytic homeostasis [104,105]. Excessive ROS inhibits the activity of three major rate-limiting enzymes, HK, PFK-1, and PK, thereby markedly impairing glycolytic flux at the early stages [106,107]. The activities of downstream enzymes, including aldolase and PGK, are also compromised, resulting in a sharp decline in ATP production that is insufficient to support OB differentiation and mineralization [108,109]. Meanwhile, mitochondrial damage impairs NADH oxidation through the ETC, leading to intracellular NADH accumulation [110]. As a consequence, LDHA continuously reduces pyruvate to lactate, resulting in lactate accumulation and microenvironmental acidosis [111]. This acidic microenvironment further inhibits the activity of HK, PFK-1, PK, and other downstream enzymes. As a result, a vicious cycle is established, leading to energy metabolic collapse, OB dysfunction, and progression of OP [112].

#### 2.2.1. Drugs Regulating LDHA

##### Oxamate (OXA)

OXA, an LDH inhibitor, has been reported to exert bone anabolic effects in murine models by improving BMD and enhancing trabecular and cortical microarchitecture. Mechanistically, OXA inhibits LDH activity, redirects glycolytic flux toward the TCA cycle, and promotes mitochondrial OXPHOS, thereby enhancing osteogenic differentiation and mineralization capacity.

However, several limitations should be considered. First, the in vivo studies are conducted predominantly in male mice and non-canonical OP models, limiting generalizability to postmenopausal OP, which primarily affects females. Second, although improvements in bone microarchitecture are reported, the absence of fracture-related endpoints limits clinical relevance. Third, while metabolic reprogramming is observed, the causal relationship between LDHA inhibition and bone formation remains insufficiently established. Additionally, potential systemic effects of LDH inhibition on other high-glycolysis tissues are not evaluated. Therefore, further studies in disease-relevant models with comprehensive functional endpoints are required to validate its therapeutic potential [113].

##### NCF

NCF has been shown to inhibit receptor activator of nuclear factor κB ligand (RANKL)-induced OC differentiation and bone resorption by suppressing glycolytic metabolism. Mechanistically, NCF downregulates key enzymes, including LDHA, HK2, and PKM2, reduces lactate production, and modulates oxidative stress-related signaling pathways.

However, several critical limitations remain. First, the evidence is restricted to in vitro studies, lacking in vivo validation and limiting physiological relevance. Second, the observed metabolic changes are largely correlative, and direct causal links to bone remodeling outcomes remain unclear. Third, the effective concentrations exceed physiologically achievable levels, raising concerns regarding translational feasibility and potential off-target effects. In addition, the evaluation is limited to osteoclastogenesis without assessment of OB-related outcomes or systemic bone remodeling balance. Therefore, the translational applicability of NCF remains uncertain [49].

##### FX11

FX11, an LDHA inhibitor, has been reported to suppress RANKL-induced OC differentiation and bone resorption by reducing intracellular lactate production and downregulating glycolytic enzymes and osteoclast-associated genes.

However, several limitations constrain the interpretation of these findings. First, the evidence is limited to in vitro systems without in vivo validation, which restricts physiological relevance. Second, LDHA inhibition may exert off-target effects on other dehydrogenases and metabolic pathways, raising concerns regarding specificity. Third, the absence of systemic toxicity assessment and long-term safety evaluation limits translational potential. Furthermore, functional endpoints related to bone remodeling, such as BMD or fracture outcomes, are not assessed. Therefore, further in vivo studies are required to establish its therapeutic value [114].

##### r-Irisin

r-irisin has been reported to modulate metabolic activity in OBs by enhancing glycolytic flux. Mechanistically, r-irisin upregulates LDHA and PDK1 expression, increases lactate production, and promotes signaling pathways associated with OB proliferation and differentiation.

However, several limitations should be considered. First, the observed metabolic effects are context-dependent, and the distinction between physiological and pathological glycolysis remains insufficiently defined. Second, inconsistencies in dosage across studies limit comparability and reproducibility. Third, the evidence is primarily derived from in vitro studies without validation in OP models. Additionally, the long-term effects of enhanced glycolysis on bone remodeling remain unclear, particularly given that excessive glycolysis is associated with pathological bone loss. Therefore, further studies are required to clarify the dose–response relationship and in vivo relevance of r-irisin [115,116].

#### 2.2.2. Drugs Regulating PFK

PFK-mediated glycolytic regulation is primarily controlled by PFK-1 and the PFK-2/PFKFB family, which modulate glycolytic flux through fructose-2,6-bisphosphate. Among these, 6-phosphofructo-2-kinase/fructose-2,6-bisphosphatase 3 (PFKFB3) is highly expressed in OBs and OCs and serves as a key regulator of glycolysis in skeletal cells [117].

Coactivator-associated arginine methyltransferase 1 (CARM1) plays a critical role in bone metabolic homeostasis by modulating glycolytic activity via PFK-1 and PFKFB3 [118]. Under pathological conditions such as OP, CARM1-mediated metabolic regulation contributes to osteometabolic imbalance.

In vitro studies demonstrate that CARM1 overexpression enhances glycolytic activity, as indicated by increased extracellular acidification rate (ECAR) and decreased oxygen consumption rate (OCR) in both OBs and OCs, whereas CARM1 deficiency exerts the opposite effects. In OBs, CARM1 promotes osteogenic differentiation, as evidenced by increased ALP activity and matrix mineralization. Notably, these effects are abolished by inhibition of PFKFB3, confirming that CARM1 regulates osteogenesis in a glycolysis-dependent manner. In contrast, CARM1 suppresses osteoclastogenesis and related gene expression in OCs.

In OVX models, CARM1 overexpression improves BMD and trabecular microarchitecture while restoring bone metabolic balance. Collectively, these findings identify the CARM1–PFKFB3 axis as a key regulatory pathway linking glycolytic reprogramming to bone remodeling [119].

##### Nicotinic Acid (NA)

NA has been reported to enhance osteogenic differentiation in OBs by activating the sirtuin 2 (SIRT2)–CCAAT/enhancer-binding protein beta (C/EBPβ)–amphiregulin (AREG) axis and PI3K/AKT signaling, leading to increased PFKFB3 expression and glycolytic activity.

However, several limitations constrain its clinical applicability. First, the effective doses used in vivo are relatively high, raising concerns regarding translational feasibility and potential adverse effects, including hepatotoxicity. Second, the evidence is limited to preclinical models, without clinical validation. Third, the reliance on glycolysis activation as a therapeutic strategy may be context-dependent, given that excessive glycolysis is associated with pathological bone loss. Furthermore, fracture-related endpoints are not evaluated. Therefore, further studies are needed to define optimal dosing and long-term safety [120].

#### 2.2.3. Drugs Regulating PGK

##### *Eucommia ulmoides* Oliver Cortex (EuOCP3)

EuOCP3 has been shown to promote OB differentiation and enhance antioxidant capacity through activation of extracellular signal-regulated kinase (ERK)/bone morphogenetic protein 2 (BMP-2)/Smad and Nrf2-related pathways, with potential involvement in PGK-related metabolic regulation.

However, several limitations should be considered. First, the use of multiple experimental models with varying dosing regimens reduces reproducibility and comparability. Second, the mechanistic link between PGK modulation and osteogenic outcomes remains insufficiently defined. Third, the absence of clinical validation limits translational relevance. Additionally, long-term safety and pharmacokinetic profiles are not reported. Therefore, further studies are required to clarify its mechanism and clinical applicability [121].

#### 2.2.4. Drugs Regulating PKM2

##### Shikonin

Shikonin has been reported to improve BMD and suppress inflammatory signaling in senile osteoporosis (SOP) models by inhibiting PKM2 activity and associated pyroptosis pathways.

However, several limitations should be acknowledged. First, the study design involves a single-dose regimen without evaluation of dose–response relationships. Second, PKM2 inhibition may exert off-target effects in multiple proliferating cell types, raising concerns regarding systemic safety. Third, the study duration is relatively short and does not capture long-term bone remodeling outcomes. Additionally, the absence of fracture-related endpoints limits clinical relevance. Therefore, further studies are required to evaluate dose optimization and long-term safety [122].

##### NCF

NCF has been shown to inhibit OC differentiation by suppressing PKM2-mediated glycolysis and modulating redox homeostasis.

However, similar to its effects on LDHA, several limitations remain. First, the evidence is restricted to in vitro systems without in vivo validation. Second, the specificity of PKM2 regulation is not fully established, and potential off-target effects are not evaluated. Third, the lack of bone-specific functional endpoints limits clinical interpretability. Therefore, further validation in disease-relevant models is required [49].

### 2.3. The TCA Cycle

The TCA cycle is the central mitochondrial hub linking glycolysis and OXPHOS, and its dysfunction in bone cells is a key contributor to OP pathogenesis via impaired energy production and redox imbalance [123,124]. Only a few TCA cycle enzymes have been investigated as potential OP therapeutic targets to date, with ACO being the most well-characterized; other enzymes (IDH, SDH) are emerging targets with underexplored clinical potential [125,126]. ACO is an iron-containing TCA cycle enzyme that catalyzes citrate-to-isocitrate conversion, and its activity is tightly linked to bone cell iron homeostasis and ROS levels—two critical factors in OP development [127].

#### 2.3.1. Drugs Regulating ACO

##### 2-Methoxyestradiol (2ME2)

2ME2, a HIF-1α inhibitor, has been reported to attenuate bone loss in OVX models; however, its therapeutic relevance should be interpreted with caution due to pharmacological and translational limitations. In vivo, 2ME2 improves bone microarchitecture and preserves bone mass, with effects comparable to sham-operated controls. Mechanistically, 2ME2 suppresses HIF-1α signaling, leading to reduced ACO activity and disruption of TCA cycle metabolism. This metabolic perturbation is associated with the induction of ferroptosis in OCs, as evidenced by increased mitochondrial iron accumulation and lipid peroxidation [128].

**Figure 2:**
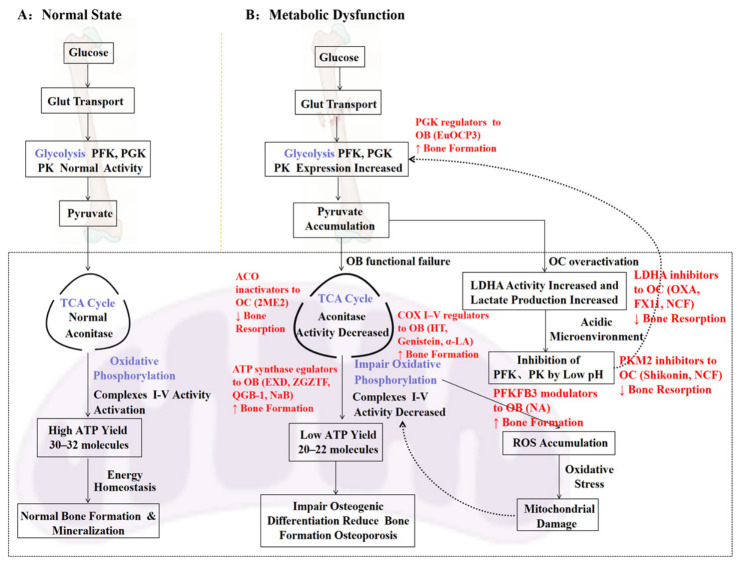
Comparison of glucose metabolism pathological changes in bone cells between normal and osteoporotic conditions. (**A**) Under normal conditions, glucose is metabolized through glycolysis and the TCA cycle to fuel OXPHOS, generating ample ATP for bone formation and resorption. (**B**) In OP, mitochondrial dysfunction and enhanced aerobic glycolysis lead to reduced ATP production, increased ROS, and impaired bone remodeling. Red indicates the treatment method.

However, several important limitations should be considered. First, the mechanistic relationship between HIF-1α inhibition, TCA cycle disruption, and ferroptosis remains largely associative, and direct causal links to bone remodeling outcomes are insufficiently established. Second, although the OVX model is widely used, it primarily reflects estrogen-deficient OP and does not capture the full spectrum of disease heterogeneity, including age-related or secondary OP. Third, 2ME2 exhibits poor oral bioavailability, systemic toxicity, and a narrow therapeutic index, which significantly limit its clinical applicability [129,130]. In addition, the absence of fracture-related endpoints and long-term safety evaluation further constrains the interpretation of its therapeutic potential. Therefore, despite its mechanistic relevance as a metabolic modulator, the translational feasibility of 2ME2 in OP therapy remains uncertain. (Figure 2).

### 2.4. Other Key Glucose-Linked Control Points in Bone Metabolism

Beyond the core metabolic pathways, several glucose-related nodes collectively contribute to the disruption of bone remodeling balance in OP by regulating the energy supply and redox balance of bone cells [131,132,133]. Among them, the pyruvate dehydrogenase (PDH)-PDK axis determines whether glycolytic products enter mitochondria for OXPHOS. In OP, the activity of this axis is abnormally elevated, which, on one hand, weakens the energy metabolism of OBs and inhibits bone formation, and on the other hand, drives OCs to rely excessively on glycolysis, thereby enhancing bone resorption [134]. Similarly, reduced activity of key TCA cycle enzymes (IDH, SDH) leads to insufficient ATP production and further exacerbates OB dysfunction [125,135,136,137]. Dysregulation of the mitochondrial pyruvate carrier (MPC) produces a bidirectional effect—suppressing OCs while promoting OB differentiation—by reducing mitochondrial energy production [138,139]. At the substrate uptake level, GLUT1/GLUT4 exhibit cell-type-specific aberrant expression: upregulation of GLUT1 in OCs fuels glycolytic bone resorption, whereas downregulation of GLUT4 in OBs limits OXPHOS and inhibits bone formation [140,141]. Furthermore, the PPP shows decreased activity in OBs, leading to reduced antioxidant capacity, while its increased activity in OCs supports their glycolytic flux. Thus, the PPP contributes to the imbalance between bone resorption and formation from both redox and metabolic flux perspectives [142,143,144]. Collectively, these nodes directly or indirectly aggravate bone remodeling imbalance in OP by perturbing bone cell energy homeostasis or redox balance. At present, only the PDK-PDH axis is supported by substantial preclinical evidence; the other nodes remain emerging or speculative targets, representing potential directions for future therapeutic interventions (Table 2).

## 3. Discussion

This review systematically synthesizes current evidence on the regulation of key enzymes involved in OXPHOS, glycolysis, and the TCA cycle in the context of OP. Metabolic reprogramming of OBs and OCs represents a central mechanistic driver of bone remodeling imbalance. Importantly, emerging evidence indicates that regulating specific metabolic nodes does not merely correct downstream phenotypic alterations but rather addresses the underlying bioenergetic dysfunction. Therefore, modulation of glucose metabolism should be regarded as a mechanistically grounded therapeutic strategy, rather than a purely adjunctive metabolic intervention. Both bone formation and bone resorption are energy-intensive but metabolically distinct processes, characterized by differential reliance on mitochondrial versus glycolytic pathways. Under pathological conditions such as estrogen deficiency or aging, suppression of OXPHOS and TCA cycle activity in OBs induces mitochondrial dysfunction and ROS accumulation [145,146]. This, in turn, drives a hierarchical metabolic shift toward glycolysis. Although this shift may transiently compensate for energy deficits, its persistence leads to reduced ATP efficiency, lactate accumulation, and microenvironmental acidosis [147]. Importantly, these metabolic alterations differentially affect OBs and OCs, ultimately disrupting signaling pathways such as AMPK and PI3K/AKT [148]. Therefore, therapeutic strategies should prioritize specific metabolic nodes according to their dominant functional role in each cell type, rather than uniformly targeting global energy metabolism.

A critical conceptual framework for interpreting the therapeutic potential of metabolic interventions hinges on recognizing fundamentally distinct metabolic phenotypes between OBs and OCs. As summarized in Table 3, quiescent OBs predominantly rely on the TCA cycle and OXPHOS to sustain bone homeostasis [149]. During differentiation, a transient moderate elevation in glycolytic flux meets the enhanced biosynthetic demands of osteoprogenitors. Subsequently, the mineralization phase reengages robust TCA cycle and OXPHOS activity to facilitate matrix deposition [140,150]. Key regulatory enzymes include COX I–V, ACO, IDH, and SDH, underscoring their pivotal roles in OB metabolic programming [151]. In contrast, resting OCs undergo dramatic glycolytic upregulation that persists through the bone resorption phase [152]. This metabolic switch supports rapid ATP generation and enables acidification of the resorption lacuna, a hallmark of OC activity [18]. Consistently, key glycolytic regulators such as LDHA, PKM2, GLUT1, and monocarboxylate transporter 4 (MCT4) are significantly upregulated during osteoclastogenesis. In estrogen-deficient postmenopausal and senile OP, OC glycolysis is further enhanced, driving excessive bone resorption. Concurrently, OBs exhibit suppressed TCA cycle and OXPHOS alongside compensatory glycolysis, leading to lactate accumulation, impaired bone formation, and disrupted bone remodeling balance. Secondary OP can arise from multiple triggers. For instance, a high-glucose environment impairs mitochondrial function by inducing ROS accumulation and reducing TCA cycle/OXPHOS efficiency, while glucocorticoids overactivate OC glycolysis to promote bone resorption [39,40,41].

Based on these cell-type-specific metabolic dependencies, a clear therapeutic hierarchy can be established. The primary therapeutic priority is the suppression of pathological glycolysis in OCs, as excessive osteoclastic activity represents the dominant driver of bone loss across most OP subtypes. Within this context, LDHA emerges as the most actionable and mechanistically central target, given its role as the terminal enzyme of glycolysis and its direct control over lactate production and metabolic flux. Pharmacological inhibition of LDHA (e.g., FX11, OXA) has consistently demonstrated the ability to suppress OC differentiation and activity without compromising OB viability. A secondary but complementary priority is the restoration of mitochondrial energy metabolism in OBs through enhancement of OXPHOS and TCA cycle activity. Targets such as COX I–V, ACO, and IDH are particularly relevant in this context, as they support ATP production and osteogenic differentiation. Compared with glycolysis inhibition in OCs, this strategy primarily exerts anabolic effects and may be more context-dependent. Collectively, this hierarchical framework underscores a key principle: anti-resorptive strategies targeting OC glycolysis (particularly LDHA) should be prioritized over pro-anabolic interventions, while combined modulation of both pathways may yield optimal therapeutic outcomes (Table 3).

This review suggests that interventional strategies can be designed according to specific cell types, metabolic pathways, and different OP subtypes. To maintain glucose metabolic homeostasis, natural compounds such as HT, genistein, and alpha-lipoic acid, as well as estrogen signaling, primarily enhance the bioenergetic output of OBs through antioxidant effects, stabilization of MMP, and potentiation of ETC activity and ATP synthase function. Notably, several TCM formulations, including EXD, ZGZTF, and QGB-1, have been shown to up-regulate ATP synthase expression and improve BMD and bone metabolism markers in both experimental and clinical studies. This reflects their holistic therapeutic approach, which systematically modulates the energy network via multicomponent, multitarget mechanisms.

Regarding the modulation of glycolytic flux, inhibitors modulating LDHA or PKM2 in OCs, such as FX11, shikonin, and NCF, effectively suppress the glycolytic metabolic reprogramming upon which OC overactivation depends, thereby reducing lactate and ROS production and specifically inhibiting bone resorption. For OBs, moderate enhancement of glycolysis-related signaling (e.g., via NA) may supply necessary energy and biosynthetic precursors for differentiation and maturation. Furthermore, regulation of key glycolytic enzymes such as PFK and PGK has shown potential to influence osteogenic differentiation by modulating the glycolysis–oxidative stress balance [153,154].

As a central metabolic hub, dysfunction of the TCA cycle is equally critical [155]. Damage to key enzymes such as ACO by ROS can significantly impair the efficiency of this cycle [156,157]. Research on 2ME2 further elucidates a novel link between metabolism, hypoxia sensing (via HIF-1α), and cell death modalities, including ferroptosis, achieving bone protection by possibly involving modulation of TCA cycle-related metabolism and induction of ferroptosis in OCs, thereby expanding the mechanistic dimension of metabolic intervention.

However, the studies summarized in this review employ a range of experimental models, each with inherent strengths and limitations. Cell-line models offer high-throughput screening capability and mechanistic insight but lack the complex cellular microenvironment and systemic regulation present in vivo. Primary cell cultures derived from rodent calvariae or bone marrow provide greater physiological relevance but are limited by donor variability and limited proliferation capacity.

In this review, OP research animal models mainly include OVX rodents, orthodontic tooth movement models, high-fat diet-induced obese models, and Glucocorticoid-Induced Osteoporosis (GIOP) models. OVX rodents are the gold standard for postmenopausal OP studies, recapitulating estrogen deficiency-driven bone loss. The GIOP model is valuable for glucocorticoid-related secondary OP but has a distinct mechanism: impaired OB differentiation, enhanced OC glycolysis, and induced OB ferroptosis. This limits its generalizability to other subtypes like postmenopausal or senile OP. Notably, diabetes is a major secondary OP contributor, characterized by high-glucose-induced OB mitochondrial damage and enhanced OC PPP activity, yet dedicated diabetic OP models are underrepresented. Moreover, most studies use young, healthy rodents, while human OP mainly affects older individuals with comorbidities such as metabolic or cardiovascular diseases. This physiological discrepancy may compromise the translational predictability of preclinical findings.

Although the preclinical landscape is promising, several critical limitations must be acknowledged to bridge the gap to clinical application. First, a significant disconnect exists between in vitro and in vivo evidence. Many studies, such as those on HT and genistein, report efficacy at concentrations (e.g., 5–100 μM) that are either at the upper limit or far exceed clinically achievable plasma levels in humans [64,68]. Supraphysiological dosing raises concerns about specificity and off-target toxicity. Second, the translatability of animal models is limited. Most in vivo studies use young, healthy rodents subjected to acute OVX, which does not fully recapitulate the chronic, multifactorial nature of age-related or postmenopausal OP in elderly humans with comorbidities. The high-fat diet model used in the NaB study is more representative of metabolic syndrome than pure OP, limiting its direct relevance [92]. Third, the potential for systemic metabolic side effects is a major concern. Regulating ubiquitous enzymes like LDHA or PKM2 with inhibitors such as FX11 or shikonin, while effective in suppressing OCs, could adversely affect other high-glycolysis tissues, including the brain, heart, and immune cells. The long-term safety of these agents on global glucose homeostasis and organ function remains largely unexplored. Fourth, the current evidence base is heavily weighted toward biomarker endpoints (e.g., BMD, ALP, TRAP) rather than hard clinical outcomes like fracture risk reduction. While BMD is a validated surrogate, fracture prevention is the ultimate goal of OP therapy. None of the clinical studies reviewed, including those on TCM formulas, were powered to detect differences in fracture incidence. This represents a critical gap. Finally, the lack of bone-specific delivery systems exacerbates the risk of off-target effects. Without a method to selectively target bone cells, systemic modulation of glucose metabolism is unlikely to be a viable long-term strategy. Future research must prioritize the development of bone-targeting moieties (e.g., bisphosphonate-conjugated nanoparticles or aptamers) to deliver metabolic modulators directly to the skeletal niche [158].

Despite these substantial limitations, the current evidence base can still be stratified into distinct translational tiers with differing levels of clinical relevance. Indeed, no intervention reviewed has demonstrated fracture risk reduction in randomized controlled trials; nevertheless, three tiers of evidence exist. The first tier comprises clinically evaluated interventions, including genistein and TCM formulations (EXD, ZGZTF, QGB-1). These represent the most advanced stage of translation, as they have been tested in human populations and demonstrate improvements in BMD, pain symptoms, and bone turnover markers. However, their clinical interpretation is constrained by consistent limitations: absence of fracture endpoints, short study durations, and frequent reliance on open-label designs, which collectively reduce the strength and generalizability of the evidence. Their safety profiles are relatively well characterized, and some agents (e.g., genistein) have been evaluated in double-blind trials, providing a solid foundation for larger definitive studies. The second tier comprises preclinically validated candidates, including OXA, NaB, shikonin, 2ME2, and NA. These agents demonstrate in vivo efficacy in established OP models (e.g., OVX or aged rodents) and provide strong mechanistic tractability by regulating defined metabolic nodes such as LDHA, PKM2, and HIF-1α. They therefore represent promising directions for therapeutic development [92,113,120,122,128]. However, their translational potential remains limited by the use of simplified animal models, lack of long-term safety evaluation, absence of fracture risk endpoints, and often insufficiently characterized dosing regimens and pharmacokinetic profiles. Several of these agents target specific metabolic nodes, enabling precise intervention and serving as tool compounds for proof-of-concept studies. The third tier consists of in vitro-only candidates (HT, NCF, FX11, α-LA, r-irisin). These are often tested at supraphysiological concentrations without in vivo validation, yet they provide the most direct evidence for cellular and molecular mechanisms, frequently at the level of individual metabolic enzymes or signaling axes. Their chemical structures are relatively simple, making them attractive leads for medicinal chemistry optimization or nanoparticle-based bone-targeted delivery. Collectively, these distinct advantages-ranging from clinical accessibility and mechanistic clarity to structural tractability-underscore the complementary value of all three evidence tiers in accelerating the translation of metabolism-targeted OP therapies, even in the face of the significant limitations outlined above.

Additionally, clinical studies included in this review are predominantly small-sample, open-label trials with a follow-up duration ranging from one to two years. While these provide valuable preliminary evidence, the absence of large-scale, double-blind, placebo-controlled randomized controlled trials with fracture outcomes represents a significant gap in the current evidence base. Furthermore, TCM formulas present a unique safety consideration due to their multi-component nature. Although the combination of multiple bioactive compounds may confer synergistic therapeutic effects, it also increases the potential for herb-drug interactions and unpredictable adverse events, particularly when used concurrently with conventional OP therapies.

**Figure 3:**
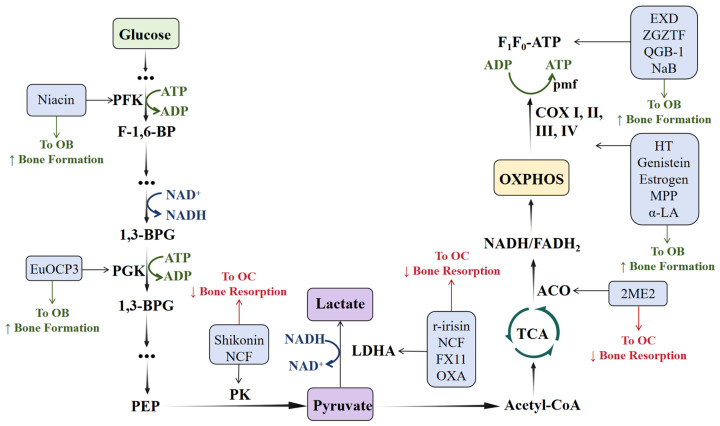
Overview of therapeutic agents targeting key glucose metabolism enzymes in OP, with a target–drug–outcome mapping.

In conclusion, modulating key glucose metabolism enzymes represents a novel therapeutic paradigm for OP, shifting the focus from “counteracting the phenotypic outcomes of dysfunctional bone cells” to “correcting the root cause of their energy metabolism imbalance” [159]. A wide range of metabolic modulators, including natural products, synthetic small molecules, and complex herbal systems, have shown preliminary biological efficacy [160]. Despite ongoing challenges in target specificity, clinical translation, and personalized application, a deeper understanding of the bone metabolic network, together with integrated novel tools such as targeted delivery systems, multi-omics analyses, and artificial intelligence, is expected to advance precise interventions regulating bone cell bioenergetics. These approaches may become an essential component of future comprehensive OP treatment strategies [161]. By rectifying energy metabolism dysfunction and the ensuing oxidative stress, the modulation of key metabolic nodes can restore bone remodeling balance [145]. Future efforts should focus on translating these promising preclinical findings into targeted clinical therapies, optimizing drug specificity and safety, and further exploring the crosstalk between metabolic pathways and other established regulatory mechanisms in skeletal biology (Figure 3).

Fracture is the most severe and direct complication of OP, representing the most critical risk indicator that requires attention in osteoporotic patients. Therefore, we conducted a focused review of the relevant literature. Through our literature investigation, we found that fracture risk is not only a clinical endpoint of OP but is also closely associated with bone cell metabolism, particularly the glycolysis process [162]. Specifically, fracture occurrence is often accompanied by alterations in bone metabolic markers and disruptions in the energy metabolism of bone cells [163]. For instance, fragility fractures represent one of the most devastating outcomes of OP. Studies have shown that fracture risk is associated with elevated bone metabolic markers (such as advanced glycation end products) and dysregulated oxidative metabolism [164]. Furthermore, recent bioinformatics analyses have revealed that glycolysis-related differentially expressed genes (GRDEGs) play a critical role in the onset and progression of OP, suggesting a potential link between glycolytic metabolism and fracture risk [165]. Therefore, as a core endpoint indicator of OP, fracture risk should be a primary focus of our research.

## 4. Conclusions

In summary, OP is increasingly regarded as a metabolic disorder characterized by impaired glucose utilization, mitochondrial dysfunction, and excessive ROS accumulation, which collectively disrupt the energy homeostasis required for coordinated OB and OC activity. Current evidence indicates that modulating the activity of key enzymes in OXPHOS, glycolysis, and the TCA cycle can effectively reprogram bone cell metabolism, restore ATP production, and rebalance bone remodeling. Natural compounds such as hydroxytyrosol, genistein, α-lipoic acid, and shikonin, along with enzyme-specific inhibitors like FX11 and NCF, have shown promising efficacy in preclinical models by improving mitochondrial function or suppressing pathological glycolysis, thereby modulating osteogenic and osteoclastic activities. In parallel, multi-component TCM formulations, including EXD, ZGZTF, and QGB-1, exert multi-target regulatory effects on metabolic pathways and have demonstrated clinical potential in improving BMD and alleviating symptoms. Specifically, LDHA or PKM2 inhibitors show particular promise for suppressing OC activity, while PFKFB3-related metabolic modulation represents a viable strategy for promoting OB differentiation. Multi-component TCM formulas with preliminary clinical evidence offer a unique opportunity for holistic metabolic intervention, though their pharmacodynamic material basis and quality standards require further elucidation. Despite these advances, challenges remain, such as limited bone specificity, incomplete mechanistic insight, and insufficient clinical translation, which hinder therapeutic application. Importantly, most interventions reviewed have not been evaluated for fracture risk reduction in randomized controlled trials, representing the most significant gap in current evidence. Future research should focus on identifying precise metabolic checkpoints in bone cells, enhancing target specificity through bone-targeted delivery systems, and integrating multi-omics approaches with high-resolution metabolic mapping. Collectively, interventions aimed at correcting metabolic dysfunction represent a mechanistically grounded and promising strategy for OP treatment. Further refinement of these approaches is expected to advance the development of next-generation, metabolism-targeted therapies capable of sustainably restoring bone homeostasis.

After the onset of OP, fracture risk increases significantly. Specifically, fracture risk is not only a clinical endpoint of OP but is also directly linked to bone cell energy metabolism, particularly the glycolytic process. Fracture occurrence is often accompanied by elevated levels of bone metabolic markers (e.g., advanced glycation end products) and dysregulated oxidative metabolism, which reflect altered glycolytic flux and mitochondrial dysfunction. To accelerate clinical translation, future research should prioritize validating metabolic biomarkers that can predict treatment response or fracture risk, including circulating lactate/pyruvate ratios, NAD^+^/NADH levels, TCA cycle intermediates, and oxidative stress markers (e.g., SOD, MDA).

## Figures and Tables

**Table 1:** Summary of Clinical Studies on TCM Formulas for OP.

Drug	Sample Size (N)	Study Design	Treatment Group	Control Group	Diagnostic Criteria	Endpoints	Adverse Events	Key Limitations	Reference
Genistein	N = 389 (treatment 195, control 194)	Randomized, double-blind, placebo-controlled	Genistein (54 mg/d) + calcium (500 mg/d) + vitamin D (400 IU/d) for 24 months	Placebo + calcium (500 mg/d) + vitamin D (400 IU/d) for 24 months	BMD at the femoral neck < 0.795 g/cm^2^	Genistein group vs. control group ① Lumbar spine BMD: 0.049 g/cm^2^ (95% CI 0.035–0.059) vs. −0.053 g/cm^2^ (95% CI –0.058 to –0.035), *p* < 0.001; ② Femoral neck BMD: 0.035 g/cm^2^ (95% CI 0.025–0.042) vs. −0.037 g/cm^2^ (95% CI −0.044 to −0.027), *p* < 0.001.	Genistein group: 19% gastrointestinal AEs; placebo: 8% (*p* = 0.002)	Lack of fracture outcomes, 19% GI adverse events (*p* = 0.002 vs. placebo) may limit tolerability, and BMD changes modestly (~0.04–0.05 g/cm^2^).	[71]
	N = 90 (genistein 30, hormone therapy 30, placebo 30)	Randomized, double-blind, placebo-controlled	Genistein (54 mg/d) for 24 months	Placebo + 17β-estradiol (1 mg/d) + norethisterone acetate for 24 months	BMD at the femoral neck < 0.795 g/cm^2^	Genistein group vs. control group ① Lumbar spine BMD: 3.0 ± 2.0% vs. 3.8 ± 2.7%, *p* < 0.001; ② Femoral neck BMD: 3.6 ± 3.0% vs. 2.4 ± 2.0%, *p* < 0.001; ③ Ward’s triangle BMD: 4.0 ± 2.7% vs. 3.0 ± 2.0%, *p* < 0.01.	Genistein: no significant differences in endometrial thickness, mammographic indicators, or hot flashes; HT group: breast tenderness, vaginal bleeding	Small sample size (N = 30 per group), no fracture data, and genistein did not relieve hot flashes (suggesting lack of systemic estrogenic effect on symptoms).	[72]
EXD	N = 90 (treatment 45, control 45)	Randomized, open-label	EXD (400 mL/d) + calcium carbonate D3 (1 tablet/d) + alfacalcidol (1 capsule/d) for 12 weeks	Calcium carbonate D3 (1 tablet/d) + alfacalcidol (1 capsule/d) for 12 weeks	Total effective rate (Expert Consensus on Prevention and Treatment of Primary OP with Chinese Medicine 2020)	EXD group vs. control group ① Visual analog scale (VAS) score: 2.10 ± 0.87 vs. 2.98 ± 1.10, *p* = 0.001; ② Procollagen type I N-terminal propeptide (PINP): 8.08 ± 11.90 vs. 45.96 ± 13.38 ng/mL, *p* = 0.0000; ③ Bone gla protein (BGP): 14.09 ± 3.97 vs. 16.14 ± 3.67 ng/mL, *p* = 0.000; ④ Serum miR-335-5p expression: 7.71 ± 1.94 vs. 1.36 ± 0.83, *p* < 0.001.	Not reported	Open-label design introduces bias, short duration (12 weeks), insufficient for BMD change, no fracture endpoints; BMD not significantly improved vs. control, and TCM syndrome-based enrollment limits generalizability.	[76]
	N = 90 (treatment 45, control 45)	Randomized, open-label	EXD (500 mL/d) + calcium carbonate D3 (1 tablet/d) + alendronate sodium (1 tablet/d)	Calcium carbonate D3 (1 tablet/d) + alendronate sodium (1 tablet/d)	Western diagnostic criteria: Guidelines for the Diagnosis and Treatment of Primary Osteoporosis (2017) TCM diagnostic criteria: Expert Consensus on the Prevention and Treatment of Primary Osteoporosis with Traditional Chinese Medicine (2020)	EXD group vs. control group ① VAS score: 2.31 ± 1.83 vs. 3.36 ± 1.94, *p =* 0.0013; ② PINP: 41.19 ± 7.81 vs. 36.37 ± 6.29 ng/mL, *p* < 0.001; ③ BGP: 17.08 ± 2.30 vs. 15.11 ± 2.40 ng/mL, *p* < 0.001; ④ β-C-terminal telopeptide of type I collagen (β-CTX): 0.366 ± 0.058 vs. 0.355 ± 0.059 ng/mL, *p* < 0.001.	Not reported	Open-label, short follow-up (12 weeks), no fracture data, BMD not reported, control group received alendronate, but no direct comparison of fracture reduction.	[80]
ZGZTF	N = 100 (treatment 50, control 50)	Randomized, open-label	ZGZTF decoction (daily, one dose daily, taken in two divided doses) for 3 months	Nilestriol tablets (2 mg/14 d) for 3 months	(1) Generalized weakness, progressive severe low back pain, fracture after minor trauma. (2) Often with spinal kyphosis. (3) X-ray shows generalized OP (spine, pelvis, proximal femur); spinal changes are typical. (4) Positive L1–L4 BMD. Reference “New Advances in the Diagnosis and Treatment of Postmenopausal Osteoporosis” in China.	ZGZTF group vs. control group ① Total effective rate (By the Nimodipine method in accordance with the Guiding Principles for Clinical Research of New Chinese Medicines): 94.0% vs. 92.0%; ② Lumbar spine BMD: 0.797 ± 0.121 vs. 0.794 ± 0.124 g/cm^2^, *p* < 0.05.	ZGZTF group: mild transient gastric discomfort; control group: no significant AEs	Open-label; short duration (3 months); no fracture outcomes; control used nilestriol, which is not a standard first-line OP therapy today, and symptom score-based efficacy subjective.	[85]
	N = 126 (treatment 63, control 63)	Randomized, open-label	ZGZTF capsules (0.9 g, 3 times/d) + teriparatide (20 μg/d, s.c.) for 6 months	Teriparatide (20 μg/d, s.c.) for 6 months	Guideline for the Diagnosis and Treatment of Primary Osteoporosis (2017)	ZGZTF group vs. control group ① Total effective rate: 92.1% vs. 79.4%, *p* < 0.05; ② L1–L4 BMD: 0.78 ± 0.11 vs. 0.72 ± 0.13 g/cm^2^, *p* < 0.05; ③ Femoral neck BMD: 0.81 ± 0.09 vs. 0.75 ± 0.11 g/cm^2^, *p* < 0.05; ④ Total hip BMD: 0.75 ± 0.10 vs. 0.71 ± 0.12 g/cm^2^, *p* < 0.05; ⑤ PINP: 171.43 ± 38.69 vs. 129.60 ± 34.81 μg/L), *p* < 0.05; ⑥ β-CTX: 0.61 ± 0.12 vs. 0.45 ± 0.11 μg/L, *p* < 0.05; ⑦ IL-33: 27.95 ± 4.07 vs. 18.14 ± 3.25 ng/L, *p* < 0.05; ⑧ IL-17: 88.45 ± 16.59 vs. 103.61 ± 21.07 ng/L, *p* < 0.05; ⑨ Insulin-like growth factor 1 (IGF-1): 142.07 ± 28.45 vs. 115.32 ± 32.06 μg/L, *p* < 0.05.	Not reported	Open-label; no blinding, no fracture data, and combination vs. teriparatide alone, lack of placebo control for ZGZTF.	[86]
	N = 86 (treatment 43, control 43)	Randomized, open-label	ZGZTF capsules (4 capsules, 3 times/d) + Sodium ibandronate injection (3 mg/3 months, i.v.) for 12 months	Sodium ibandronate injection alone (3 mg/3 months, i.v.) for 12 months	Guideline for the Diagnosis and Treatment of Primary Osteoporosis (2017)	ZGZTF group vs. control group ① Total effective rate: 95.35% vs. 81.39%, *p* < 0.05; ② Femoral neck BMD: 0.96 ± 0.48 vs. 0.87 ± 0.32 g/cm^2^, *p* < 0.05; ③ Lumbar spine BMD: 0.98 ± 0.22 vs. 0.77 ± 0.41 g/cm^2^, *p* < 0.05; ④ Femoral trochanter BMD: 0.82 ± 0.70 vs. 0.58 ± 0.33 g/cm^2^, *p* < 0.05; ⑤ SF-36 score: 80.67 ± 6.37 vs. 67.28 ± 5.19, *p* < 0.05; ⑥ IL-6: 21.87 ± 4.16 vs. 35.29 ± 6.45 ng/L, *p* < 0.05; ⑦ IGF-1: 159.28 ± 15.49 vs. 113.64 ± 13.53 μg/L, *p* < 0.05; ⑧ TNF-α: 25.38 ± 2.76 vs. 40.18 ± 4.59 ng/L, *p* < 0.05; ⑨ PINP: 65.48 ± 14.05 vs. 52.75 ± 12.20 mg/L, *p* < 0.05.	Not reported	Open-label, no blinding, no fracture outcomes, and small sample size (N = 43/group).	[87]
	N = 108 (treatment 54, control 54)	Randomized, open-label	ZGZTF capsules (4 capsules, 3 times/d) + Raloxifene Hydrochloride Tablets (60 mg/d) for 8 weeks	Hydrochloride Tablets alone for 8 weeks	Guideline for the Diagnosis and Treatment of Osteoporosis in the Elderly in China (2018)	ZGZTF group vs. control group ① Total effective rate: 98.15% vs. 81.48%, *p* < 0.05; ② BMD: 0.91 ± 0.17 vs. 0.74 ± 0.23 g/cm^2^, *p* < 0.05; ③ Sclerostin (SOST): 10.19 ± 0.77 vs. 13.28 ± 0.84 pg/mL, *p* < 0.05; ④ IL-6: 11.34 ± 2.25 vs. 34.21 ± 4.16 pg/mL, *p* < 0.05; ⑤ E_2_: 77.39 ± 7.48 vs. 54.72 ± 8.63 pmol/L, *p* < 0.05; ⑥ Serum calcium: 2.62 ± 0.61 vs. 2.23 ± 0.48 mmol/L, *p* < 0.05; ⑦ Serum phosphorus: 1.29 ± 0.21 vs. 1.71 ± 0.34 mmol/L, *p* < 0.05; ⑧ ALP: 48.79 ± 4.35 vs. 83.14 ± 5.24 U/L, *p* < 0.05; ⑨ Adverse reaction rate: 5.56% vs. 16.67%, *p* < 0.05.	Adverse reaction rate: 5.56% in combination group vs. 16.67% in control (*p* < 0.05); specific events include nausea, flushing, headache, and leg cramp.	Very short duration (8 weeks) and BMD change unlikely, open-label, no fracture data, high dropout rate not mentioned, and adverse events lower in combination but not placebo-controlled.	[88]
QGB-1	N = 46 (treatment 23, control 23)	Prospective, randomized, open-label	QGB-1 decoction (1 dose/d) + calcium D3 (600 mg/d) + calcitriol, alendronate (70 mg/week) for 3 months	Calcium D3 (600 mg/d) + calcitriol (0.5 μg/d), alendronate (70 mg/week for 3 months)	Refer to the diagnostic criteria for hip osteoarthritis, old femoral neck fractures, and femoral head necrosis in “Practical Orthopedics” in China.	QGB-1 group vs. control group ① BMD: 0.751 ± 0.108 vs. 0.689 ± 0.078 g/cm^2^, *p* < 0.05; ② ROI 1 BMD: 0.619 ± 0.149 vs. 0.504 ± 0.102 g/cm^2^, *p* < 0.05; ③ ROI 4 BMD: 1.330 ± 0.213 vs. 1.185 ± 0.217 g/cm^2^, *p* < 0.05; ④ ROI 6 BMD: 1.280 ± 0.217 vs. 1.096 ± 0.166 g/cm^2^, *p* < 0.05; ⑤ ROI 7 BMD: 1.052 ± 0.172 vs. 0.931 ± 0.172 g/cm^2^, *p* < 0.05; ⑥ BMD loss rate: 12.0% vs. 27.1%, *p* < 0.05.	Not reported	Very small sample size (N = 23/group), open-label, short follow-up (3 months), no fracture outcomes, and surrogate endpoint (periprosthetic BMD loss rate) not a validated OP treatment endpoint.	[90]

**Table 2:** Summary of pharmacological interventions targeting glucose metabolic processes (OXPHOS, glycolysis, and the TCA cycle) in bone cells and osteometabolic disease models.

Glucose Metabolism Process	Enzyme	Drug	Types	Model	Doses/ Duration	Effects/Mechanism	Evidence Strength	Key Limitations	Reference
OXPHOS	COX I–IV	HT	In vitro	MC3T3-E1 cells with 0.75 mM H_2_O_2_ for 6 h	Pretreated with 5 μM for 1 h	Attenuates apoptosis and mtROS generation, restores MMP, increases ATP production, restores the activities of mitochondrial COX I, II, III, and IV, increases the phosphorylation levels of AKT and GSK3β, and regulates OPA1 cleavage.	B1: In vitro studies	Only in vitro, chronic in vivo efficacy unknown, and concentration used (5 μM) is at the upper limit of the human plasma range.	[64]
	COX I/II	Genistein	In vitro	Rat cranial OBs and human U-2 OS cells	25, 50, 75, and 100 μM for 24 h, or 100 μM for 6, 12, 18, and 24 h	Increases ERα levels, mitochondrial COX I/II gene and protein expression, elevates MMP and ATP levels.	B1: In vitro studies	In vitro only, concentrations used (25–100 μM) far exceed clinical peak plasma levels (1–5 μM), and raise specificity concerns.	[68]
	COX I/II	MPP	In vitro	Primary calvarial OBs from 3-day-old Wistar rats	25, 50, and 100 μM for 24 h	Attenuates estrogen-induced ERα translocation and ATP levels, decreases BMP-6 and COLI mRNA expression, and COX I/II mRNA expression, inhibits the differentiation and mineralization of primary calvarial OBs.	B1: In vitro studies	In vitro tool compound only, not a therapeutic candidate, used to model estrogen deficiency, and not to treat OP.	[74]
	COX IV	α-LA	In vitro	50 μM AMA-induced MC3T3-E1 cells for 24 h	Pretreated with 10, 50, and 100 μM for 12 h	Inhibited AMA-induced cytotoxicity, cell death, OB dysfunction, and ROS production, restores phosphorylated AKT levels, inhibits cytochrome C release and caspase-3 activation.	B1: In vitro studies	Only in vitro; uses acute mitochondrial toxin (AMA, 50 μM) which does not replicate chronic, low-grade dysfunction of aging/postmenopausal OP; no in vivo bone outcome data.	[75]
	F_1_F_o_-ATP	EXD	In vitro	OC from RAW264.7 cells with 25 ng/mL RANKL and 30 ng/mL Macrophage colony-stimulating factor (M-CSF) for 72 h, and UMR-106 cells	50, 100, and 200 μg/mL for 48 h, 72 h, and 14 d	Promotes OB bone nodule formation, proliferation, ALP activity, and bone matrix mineralization, increases the expressions of ATP synthase, aldolase A, and GDI-α, inhibits OC TRAP activity and bone resorption pit formation, reduces calnexin expression, and upregulates the expressions of vimentin, PDI, and alpha-fetoprotein.	B1: In vitro studies	Only in vitro studies, with no in vivo validation.	[77]
	F_1_F_o_-ATP	ZGZTF	In vivo	SD rat model of OVX	0.6 mL aqueous phase + 0.4 mL oily phase/100 g body weight for 13 weeks (i.g.)	Enhances ATP production, increases the expression of CK A, ferritin, and fructose-bisphosphate aldolase A, and decreases the expression of secretogranin II.	B2: In vivo studies	The dosage is unclear, no standardized administration protocol, clinical studies open-label, no fracture data, and potential herb-drug interactions not evaluated.	[84]
	F_1_F_o_-ATP	QGB-1	In vivo	SD rat model of OVX	20 weeks	Enhances the expression of lactoferrin light chain, PK isozyme, and coronin, decreases the expression of enolase, ATP synthase, troponin, CK isozyme, phosphoglycerate mutase, and myosin. Lactoferrin light chain and troponin are associated with apoptosis; PK isoenzyme, ATP synthase, CK isoenzyme, and phosphoglycerate mutase are related to energy metabolism.	B2: In vivo studies	Only BMD and a few protein markers assessed; no cellular or molecular mechanism in vivo.	[90]
	F_1_F_o_-ATP	NaB	In vivo	High-fat diet-induced obese SD rat model	4% for 12 weeks	Reverses bone loss and body weight gain, enhances ALP activity and mineralization, increases ATP production, Mn-SOD, CAT, and GSH-Px activities, decreases the level of acetyl-CoA, ROS, and NADH/NAD^+^ ratio, and up-regulates Nrf2/GSK-3β signaling and the expression of Peroxisome proliferator-activated receptor gamma coactivator 1-alpha (PGC-1α) and Mitochondrial transcription factor A (TFAM).	B3: Mixed preclinical	High-fat diet model not a canonical OP model, dosage reported only as 4% (m/v), not absolute mg/kg, in vitro concentrations (0.1–0.5 mM) showed benefit but 0.75 mM was ineffective, indicating a narrow therapeutic window.	[92]
			In vitro	1 mM H_2_O_2_-stimulated MC3T3-E1 subclone 14 preosteoclast cells for 1 h	Pretreated with 0.1, 0.3, 0.5 or 0.75 mM for 72 h				
Glycolysis	LDHA	r-irisin	In vitro	MC3T3-E1 cells	1, 10, and 100 nM for 24 h	Promotes proliferation and differentiation of OBs, up-regulates RUNX2, osterix (Osx), ALP, and collagen type I alpha 1 (Col1a1), enhanced the expression levels of LDHA and PDK1, increased lactate levels, succinate, fumarate, malate, and Uncoupling protein 2 (Ucp2) mRNA levels Decreases citrate, isocitrate, 2-ketoglutarate, and the levels of ATP5A, MTCO1, and NDUFB8 of OXPHOS.	B1: In vitro studies	Dosage administration was inconsistent, and no standardized criteria were applied throughout the study.	[116]
	LDHA	FX11	In vitro	Bone marrow cells from the bone marrow cavity in 8-week-old male C57BL/6 N mice with 25 ng/mL RANKL and 30 ng/mL M-CSF	20, 30, 40, 50 μM	Decreases intracellular lactate concentrations, inhibits OC formation, decreases expression levels of GLUT1, glyceraldehyde-3-phosphate dehydrogenase (GAPDH), decreased the level of cathepsin K and OC bone-resorbing activity.	B1: In vitro studies	Only in vitro studies, with no in vivo validation.	[114]
	LDHA	OXA	In vitro	C3H10T1/2 cells	1 mM	Increases bone biomechanical strength, mineral apposition rate (MAR) and bone formation rate (BFR), decreases body fat percentage, increases lean mass and BMD, inhibits LDH activity, improves cortical thickness (Cort Th), cortical area (Cort Area), and cortical BMD (Cort BMD).	B3: Mixed preclinical	Only male healthy mice used, not a canonical OP model (e.g., OVX); lack of female data limits generalizability to postmenopausal OP.	[74]
			In vivo	Young and aged C57BL/6J male mice	100 mg/kg				
	LDHA/PK	NCF	In vitro	Raw264.7 cells	30 μM for 5 d	Inhibits OC differentiation, bone resorption capacity, and ROS production, reduces HK2, PKM2, and LDHA expression, decreases extracellular acid rate, and increases oxygen consumption.	B1: In vitro studies	Only in vitro studies, with no in vivo validation, only osteoclastogenesis assessed but no OB or bone formation data.	[49]
	PFK	NA	In vitro	MC3T3—E1 cells	50, 100, 200 μM for 7 d	Mitigated bone loss, increased BMD, bone volume/total volume (BV/TV), trabecular thickness (Tb.Th) and trabecular number (Tb.N), enhanced OB differentiation, promoted bone formation, upregulated osteocalcin (OCN), osteopontin (OPN), RUNX2, AREG, PFKFB3 and PFK, activated PI3K-AKT signaling pathway, increased the innate interaction between C/EBPβ and SIRT2, decreased trabecular separation (Tb.Sp) and CT. Po.	B3: Mixed preclinical	Lack of integration with clinical trials, OVX mouse model only (no aged or male models), dose (300 mg/kg/d) is high and may not be translatable to humans, and no fracture or long-term safety data.	[120]
			In vivo	OVX C57BL/6 Mouse Model	300 mg/kg/day for 2 months				
	PGK	EuOCP3	In vitro	MC3T3-E1 cells with 4 µM Dex for 24 h	10 or 20 μg/mL for 2 h	Increases Tb.BV/TV, Tb.Th, Tb.BMD, Ct.BV, Ct.Th, and Ct.Ar/Tt.Ar, decreases Tb.BS/BV, upregulates RUNX2, Osx, PICP, OPN, and OCN levels, reduces CTX-I, TRAP, CTSK, and nuclear factor of activated T cells c1 (NFATc1) levels, enhances X-ray absorption in trabecular bone, Cort Th, mineralized bone area, and OB number and area, decreases OC number and area, elevates serum glycerate levels, modulates glycine, serine, and threonine metabolism, reduces serum creatine, PGK, ROS, and MDA levels, and activates the Nrf2 and BMP-2/Smad signaling pathways.	B3: Mixed preclinical	Lack of integration with clinical trials and multiple models used (Dex-induced, orthodontic tooth movement) with variable doses (100–500 mg/kg).	[121]
			In vivo	Orthodontic tooth movement model with 30 mg/kg Dex-Induced C57BL/6 Mice for 6 months	500 mg/kg for 8 weeks				
			In vivo	30 mg/kg Dex-Induced C57BL/6 male mice	100, 300 mg/kg for 7 weeks				
	PK	Shikonin	In vivo	Senile OP C57BL/6 mouse model	2 mg/kg/d for 6 weeks	Suppresses Interleukin-1β (IL-1β), Interleukin-18 (IL-18), and TNF-α levels, decreases PKM2 activity, upregulates RUNX2, and downregulates NOD-like receptor protein 3 (NLRP3), Apoptosis-associated speck-like protein containing a CARD (ASC), and cleaved gasdermin D (GSDMD).	B2: In vivo studies	Single-dose study, no dose–response assessment, short duration only 6 weeks, senile OP mouse model used but no female or postmenopausal data, and PKM2 inhibition may have off-target effects on other proliferating cells.	[122]
The TCA cycle	ACO	2ME2	In vivo	C57BL/6 mice model of OVX	75 mg/kg for 4 weeks	Improves bone mass and microarchitecture, restores autophagy flux and ferritinophagy, alters iron homeostasis and lipid peroxidation, inhibits the expression of HIF-1α.	B2: In vivo studies	No dosage frequency is indicated and lack of integration with clinical trials.	[122]

For Evidence Strength column, we based on [A Critical Appraisal of Off-Label Use and Repurposing of Statins for Non-Cardiovascular Indications: A Systematic Mini-Update and Regulatory Analysis] have carried out classify (B1) in vitro studies, (B2) in vivo studies, and (B3) mixed preclinical.

**Table 3:** Integrated conceptual framework of glucose metabolism regulation in OP.

Glucose Metabolism Regulation: A Cell-Type-Specific Framework			
		OB	OC
Physiological conditions	Resting state	OXPHOS dominant	Low metabolic activity
	Activation/Differentiation state	↑ OXPHOS + ↑ TCA cycle + Glycolysis upregulation	↑ Glycolysis (Warburg-like)
	Functional state	Mineralization: OXPHOS and TCA cycle dominant	Bone resorption: ↑ Glycolysis + ↑ PPP
	Primary energy demands	Collagen synthesis, matrix mineralization	Acidification, protease secretion
	Key enzymes	COX I–V, ACO, IDH, SDH	LDHA, PKM2, GLUT1, MCT4
**Postmenopausal OP**		**Senile OP**	**Secondary OP**
**Estrogen deficiency**		**Aging-related metabolic decline**	**Common triggers: glucocorticoids, diabetes mellitus, etc.**
OC: Excessive enhancement of glycolysis (Warburg-like), increased activation OB: ↓ Mitochondrial function, ↓ bone formation → Bone resorption > Bone formation		OB: ↓ OXPHOS, insufficient energy supply, reduced differentiation capacity OC: Relatively increased basal metabolic activity → Negative bone remodeling balance	Glucocorticoids: Inhibit OB differentiation Enhance OC glycolysis Induce ferroptosis in OB Diabetes mellitus: High glucose environment → OB mitochondrial damage ↑ PPP in OC, ↑ acidification capacity → Imbalance of metabolic regulatory networks

## Data Availability

No new data were created or analyzed in this study. Data sharing is not applicable to this article.

## Abbreviations

The following abbreviations are used in this manuscript:

OP	Osteoporosis
ATP	Adenosine triphosphate
TCA cycle	Tricarboxylic acid cycle
OXPHOS	Oxidative phosphorylation
ROS	Reactive oxygen species
PDK	Pyruvate dehydrogenase kinase
PDH	Pyruvate dehydrogenase
PDC	Pyruvate dehydrogenase complex
HK	Hexokinase
LDHA	Lactate dehydrogenase A
OB	Osteoblast
OC	Osteoclast
PFK	Phosphofructokinase
PGK	Phosphoglycerate kinase
PK	Pyruvate kinase
TCM	Traditional Chinese medicine
NCF	Nuciferine
OCR	Oxygen consumption rate
ECAR	Extracellular acidification rate
ETC	Electron transport chain
PMF	proton motive force
MMP	Mitochondrial membrane potential
mPTP	mitochondrial permeability transition pore
AKT	Protein Kinase B
GSK3β	Glycogen synthase kinase 3β
ALP	Alkaline phosphatase
ERα	Estrogen receptor α
COX	Complexes
BMD	Bone mineral density
PINP	Procollagen type I N-terminal propeptide
BMP-6	Bone morphogenetic protein 6
α-LA	α-lipoic acid
AMA	Antimycin A
PI3K	Phosphoinositide 3-kinase
EXD	Er-Xian Decoction
TRAP	Tartrate-resistant acid phosphatase
VAS	Visual Analog Scale
ZGZTF	Zhuanggu Zhitong Formula
OVX	Ovariectomized
BMC	Bone mineral content
CK	Creatine Kinase
QGB-1	Qianggu Bao No. 1
NaB	Sodium butyrate
BV/TV	Bone volume/total volume
Tb.Sp	Trabecular separation
Tb.N	Trabecular number
Tb.Th	Trabecular thickness
MDA	Malondialdehyde
MPP	Methyl-piperidino-pyrazole
GLUT	Glucose transporter
GAPDH	Glyceraldehyde-3-phosphate dehydrogenase
MCT4	Monocarboxylate transporter 4
OXA	Oxamate
MAR	Mineral apposition rate
BFR	Trabecular thickness
CARM1	Coactivator-associated arginine methyltransferase 1
NA	Nicotinic acid
RUNX2	Runt-related transcription factor 2
OCN	Osteocalcin
OPN	Osteopontin
EuOCP3	*Eucommia ulmoides* Oliver cortex
COL1	Type I collagen
SOP	Senile osteoporosis
IL-1β	Interleukin-1β
IL-18	Interleukin-18
NLRP3	NOD-like receptor protein 3
GSDMD	Gasdermin D
NFATc1	Nuclear factor of activated T cells c1
ACO	Aconitase
2ME2	2-Methoxyestradiol
GSH	Glutathione
IDH	Isocitrate dehydrogenase
SDH	Succinate dehydrogenase
OGDH	α-Ketoglutarate dehydrogenase
MPC	Mitochondrial pyruvate carrier
PPP	Pentose phosphate pathway
GRDEGs	Glycolysis-related differentially expressed genes
GIOP	Glucocorticoid-Induced Osteoporosis
TFAM	Mitochondrial transcription factor A
Cort Th	Cortical Thickness
Cort Area	Cortical Area
Cort BMD	Cortical Bone Mineral Density
BGP	Bone Gla Protein
β-CTX	β-C-terminal telopeptide of type I collagen
SOST	Sclerostin
IGF-1	Insulin-like growth factor 1
AMPK	AMP-activated protein kinase
Nrf2	Nuclear factor erythroid 2-related factor 2
ERK	Extracellular signal-regulated kinase
HIF-1α	Hypoxia-inducible factor 1-alpha
SOD	Superoxide dismutase
CAT	Catalase
RANKL	Receptor activator of nuclear factor κB ligand
M-CSF	Macrophage colony-stimulating factor
BMP-2	Bone morphogenetic protein 2
PFKFB3	6-phosphofructo-2-kinase/fructose-2,6-bisphosphatase 3
PGC-1α	Peroxisome proliferator-activated receptor gamma coactivator 1-alpha
Ucp2	Uncoupling protein 2
Osx	Osterix
SIRT2	Sirtuin 2
C/EBPβ	CCAAT/enhancer-binding protein beta
AREG	Amphiregulin
ASC	Apoptosis-associated speck-like protein containing a CARD
